# Osteopontin enhances the effect of treadmill training and promotes functional recovery after spinal cord injury

**DOI:** 10.1186/s43556-023-00154-y

**Published:** 2023-11-28

**Authors:** Yunhang Wang, Hong Su, Juan Zhong, Zuxiong Zhan, Qin Zhao, Yuan Liu, Sen Li, Haiyan Wang, Ce Yang, Lehua Yu, Botao Tan, Ying Yin

**Affiliations:** 1 Department of Rehabilitation Medicine, The Second Affiliated Hospital of Chongqing Medical University, Chongqing, 400010 China; 2 Department of Rehabilitation, Zhejiang University School of Medicine Second Affiliated Hospital, 88 Jiefang Road, Hangzhou, Zhejiang 310009 China; 3 State Key Laboratory of Trauma, Burns and Combined Injury, Department of Special War Wound, Daping Hospital, Army Medical University, Chongqing, 400042 China

**Keywords:** Osteopontin, Treadmill training, Functional recovery, Spinal cord injury

## Abstract

In this study, we examined the combined impact of osteopontin (OPN) and treadmill training on mice with spinal cord injury (SCI). OPN was overexpressed by injecting AAV9-SPP1-GFP into the sensorimotor cortex, followed by a left incomplete C5 crush injury two weeks later. Mice (Ex or Ex + OPN group) were trained at 50% maximum running speed for 8 weeks. To analyze the effects, we used biotinylated dextran amine (BDA) for tracing the corticospinal tract (CST) and performed Western blotting and immunohistochemical methods to assess the activation of the mammalian target of rapamycin (mTOR). We also examined axonal regeneration and conducted behavioral tests to measure functional recovery. The results demonstrated that treadmill training promoted the expression of neurotrophic factors such as brain-derived neurotrophic factor (BNDF) and insulin-like growth factor I (IGF-1) and activated mTOR signaling. OPN amplified the effect of treadmill training on activating mTOR signaling indicated by upregulated phosphorylation of ribosomal protein S6 kinase (S6). The combination of OPN and exercise further promoted functional recovery and facilitated limited CST axonal regeneration which did not occur with treadmill training and OPN treatment alone. These findings indicate that OPN enhances the effects of treadmill training in the treatment of SCI and offer new therapeutic insights for spinal cord injury.

**Supplementary Information:**

The online version contains supplementary material available at 10.1186/s43556-023-00154-y.

## Introduction

Spinal cord injury (SCI) is a devastating central nervous system (CNS) condition that often leads to the disruption of neural circuitry and connectivity, resulting in permanent functional deficits and affecting millions of individuals worldwide [1, 2]. Not only does SCI impact the quality of patients’ lives, but it also places significant burdens on both families and society at large. Over the past several decades, significant progress has been made in our understanding of the underlying mechanisms of SCI treatment [3–5]. Nevertheless, despite these strides, there remain no cures available for SCI patients. It is urgent to develop therapeutic strategies aimed at enhancing functional recovery and alleviating the associated deficits.

Rehabilitation is considered a beneficial therapeutic strategy [6]. Previous clinical studies have reported that rehabilitative training increases physical activity levels and enhances the quality of life in people with SCI [7–11]. The mechanisms contributing to recovery have been partially demonstrated by facilitating synaptic plasticity [12, 13], promoting the expression of neurotrophins such as brain-derived neurotrophic factor (BDNF) and insulin-like growth factor 1 (IGF-1) [14–17], and then activating the downstream signaling pathway such as mTOR [14, 18, 19]. However, previous studies suggest that rehabilitative training alone have cannot promote regrowth of the CST axons [20]. It worth noting that the regrowth of CST axons plays a pivotal role in facilitating functional recovery [21].

Generally speaking, neurotrophins treatment is a promising strategy and it has been confirmed that IGF-1 or BDNF enhances the extent and rate of murine corticospinal motor neuron axon outgrowth in vitro [22]. Although treadmill training promotes BDNF and IGF-1 expression, it has rarely been reported that treadmill training or other rehabilitative training alone could promote CST axonal regeneration. This may be related to the reduced responsiveness of neurons to these neurotrophins, potentially stemming from the post-injury downregulation of neurotrophins receptors [23, 24]. Recently, there has been a hypothesis suggesting that osteopontin (OPN) may enhance neuronal responses to neurotrophins [25]. OPN which is encode by Secreted phosphoprotein 1 (SPP1), also known as bone sialoprotein 1and Eta-1 protein, exists in different tissues. As a multifunctional protein, OPN plays an important role in the process of inflammation, cell viability, and wound healing [26]. It was found that co-expression of insulin-like growth factor 1 (IGF-1) and OPN induces robust regrowth of retinal axons but without visual function recovery [27]. In spinal cord injury, recent studies also demonstrated that a combination of OPN and IGF-1 promotes CST regeneration while axonal regeneration did not occur with IGF-1 or OPN treatment alone [25].

Considering the importance of OPN in contributing to CST regeneration and the promoting effect of exercise in neurotrophins, we asked whether a combination of treadmill training and OPN could promote CST regrowth and functional recovery in clinically relevant injury models. We observed that treadmill training promoted neurotrophins expression and activated mTOR signaling after spinal cord injury, although there was no CST regeneration. Then, we tested the effect of the combination strategy of treadmill training and OPN. We discovered that the combination strategy further promoted functional recovery and limited CST regrowth. Our results reveal a possibly translatable strategy for clinical use and provide a new view of potential therapeutic strategies for SCI patients in the future.

## Result

### Treadmill training promoted the expression of BDNF and IGF-1 after spinal cord injury

**Fig. 1:**
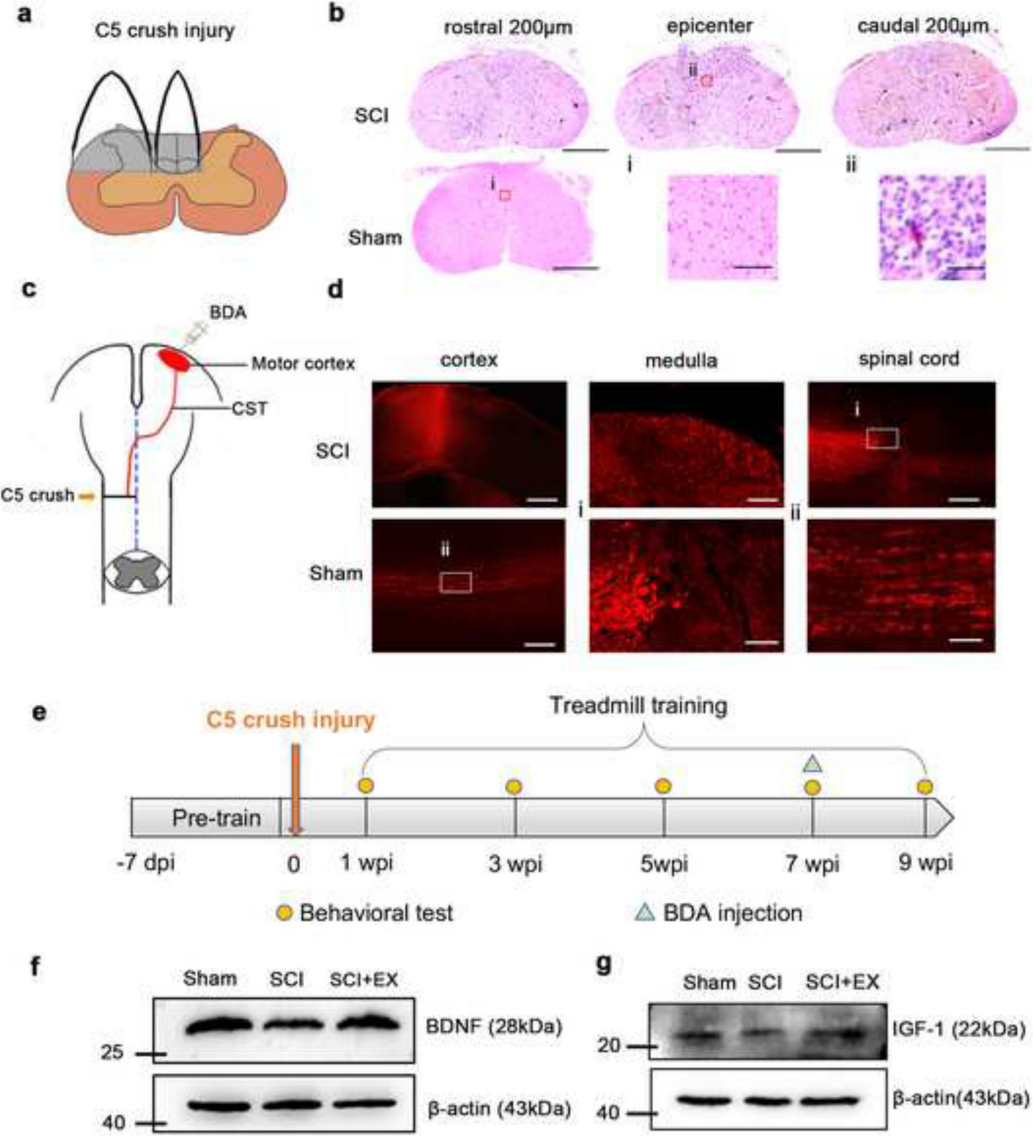
Treadmill training enhanced expression of BDNF and IGF-1 after spinal cord injury. **a** Illustration of the incomplete C5 crush. **b** Representative transverse spinal cord graphs of HE staining in the SCI and Sham group. Bar = 500 μm. (i) (ii) magnified images of the two groups. Bar = 50 μm. **c** Illustration of BDA injection. **d** Representative fluorescence images of the transverse cortex section (left), transverse medulla section (middle), and longitudinal spinal cord section (right) in the SCI group and longitudinal spinal cord section in the Sham group. Bar = 500 μm (left), 100 μm (middle), 200 μm (right). (i) (ii) magnified images of spinal cord in SCI and Sham groups. Bar = 20 μm. **e** Illustration depicting the experimental timeline I. **f** The western blot results of the cortex in the Sham (*n* = 5), SCI (*n* = 5), and SCI + EX (*n* = 5) group showing the expression of BDNF. **g** The western blot results of the cortex in the Sham, sci, and sci + ex groups showing the expression of IGF-1. Data are presented as the means ± SEMs. One-way ANOVA with Bonferroni’s post hoc analysis. *, *p* < 0.05

The predominant form of traumatic spinal cord injury is often cervical injury [28]. Hence, we utilized an animal model involving cervical injury to investigate the characteristics exhibited by adult mice following the fifth cervical spinal cord crush (C5 crush) (Fig. 1a). Histological examinations through HE staining revealed disrupted spinal cord structures, blurred demarcation between gray and white matter, and infiltration of inflammatory cells at the epicenter (Fig. 1b). The coronal section of the spinal cord in the HE staining image similarly showcased structural disruption and inflammatory cell infiltration (Fig. S1a). To assess the efficacy of incomplete C5 injury, we employed anterograde tracing. Two weeks before the mice were sacrificed, BDA was injected into the sensorimotor cortex to trace the CST axon (Fig. 1c). The BDA-labeled CST axons halted at the lesion site, confirming the success of incomplete C5 injury (Fig. 1d). Retrograde tracing with Mini ruby further validated this success (Fig. S1b-e). The Basso mouse scale (BMS) [29] score exhibited a significant reduction on day 1, recovering to normal levels within the subsequent week, indicating minimal impact on locomotion due to the incomplete C5 crush (Fig. S1f). In addition, Lesion area quantification (Fig. S2) indicated no substantial variation among individuals.

Next, we probed the impact of treadmill training on neurotrophic factor expression. The experimental timeline is detailed in Fig. 1e. Consistent with previous studies [30–32], 8 weeks of treadmill training postinjury significantly promoted the expression of BDNF. Furthermore, the SCI + EX group had an enhanced expression level of IGF-1 (SCI vs. SCI + EX, *p* = 0.04) (Fig. 1f-g, Fig. S3a-b). This observation showed that 8 weeks of treadmill training after SCI promoted the expression of BDNF and IGF-1.

### Activation of mTOR signaling in the sensorimotor cortex after treadmill training

**Fig. 2:**
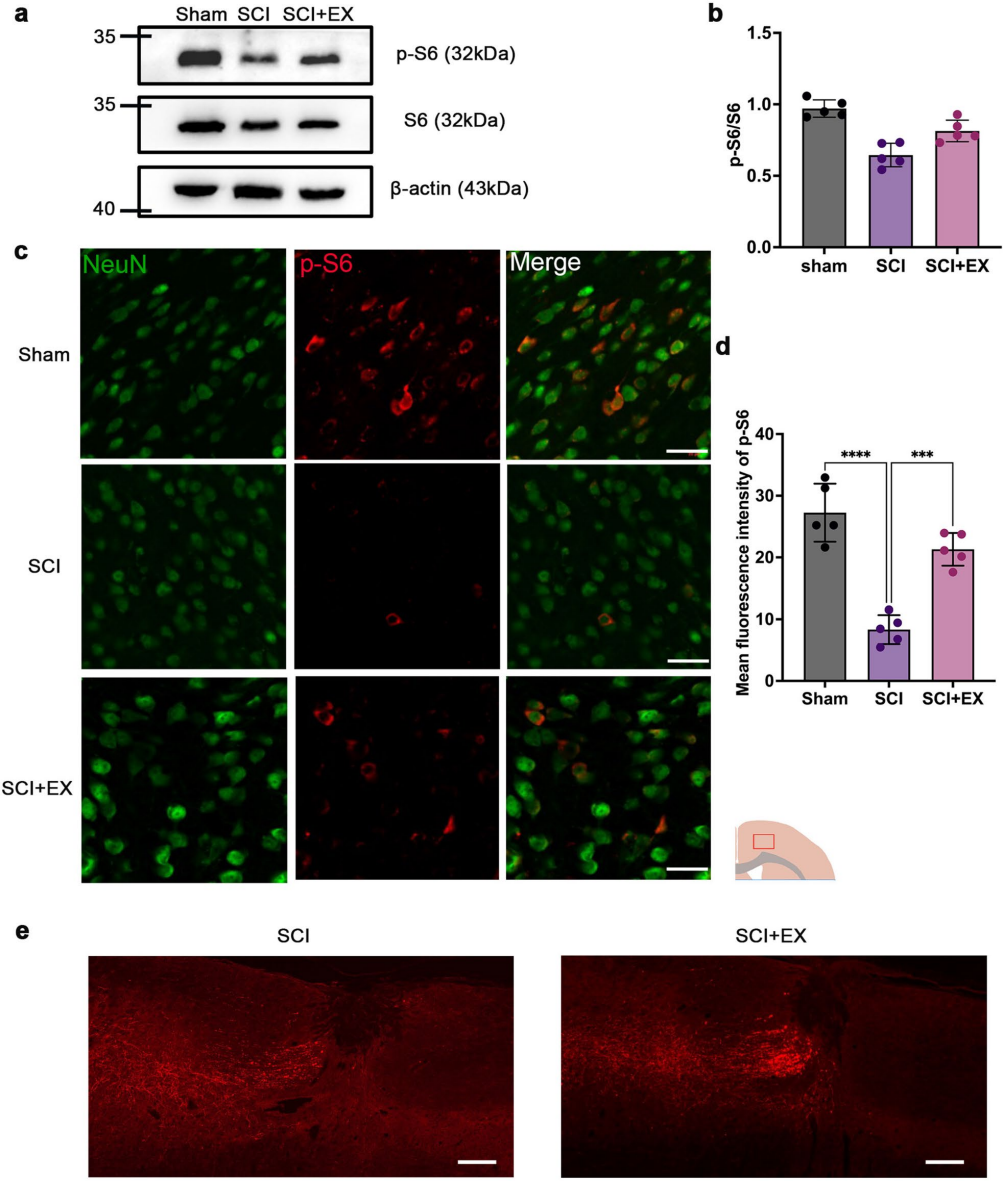
Treadmill training activated mTOR signaling without axonal regeneration. **a** The western blot results of the Sham (*n* = 5), SCI (*n* = 5), and SCI + EX (*n* = 5) groups showing the expression of p-S6 and S6. **b** Quantitative analysis of the p-S6/S6. **c** Representative fluorescence images of transverse brain sections from the Sham (*n* = 5), SCI (*n* = 5), and SCI + EX (*n* = 5) groups showing NeuN (green) and p-S6 (red). Bar = 20 μm. The illustration denotes the origin of the images captured from the cortex. **d** Quantification of the p-S6 mean fluorescence intensity. **e** Representative fluorescence images of C5 segment spinal cord sagittal sections for the SCI and SCI + EX groups. Bar = 200 μm

Previous studies have shown the significance of the CST in driving functional recovery, while mTOR signaling stands out as one of the pivotal intrinsic regenerative pathways responsible for axonal regeneration and sprouting following various central nervous system (CNS) injuries [21, 33, 34]. Therefore, we investigated the activation level of mTOR signaling and functional recovery after treadmill training. Our Western blot analysis suggested that the p-S6 level was downregulated after SCI, and treadmill training significantly upregulated the phosphorylation of S6 compared to the SCI group (SCI vs. SCI + EX, *p* = 0.044) (Fig. 2a-b). Consistently, the immunofluorescence results in the sensorimotor cortex also suggested that treadmill training significantly enhanced p-S6 expression compared to that in the SCI group (Fig. 2c-d). However, there was no axon regenerating and innervating the spinal cord caudal to the lesion (Fig. 2e). Collectively, treadmill training promoted BDNF and IGF-1 expression and activated mTOR signaling but failed to promoted CST axonal regeneration.

### The potential roles of OPN in exercise-induced repair in spinal cord injury

**Fig. 3:**
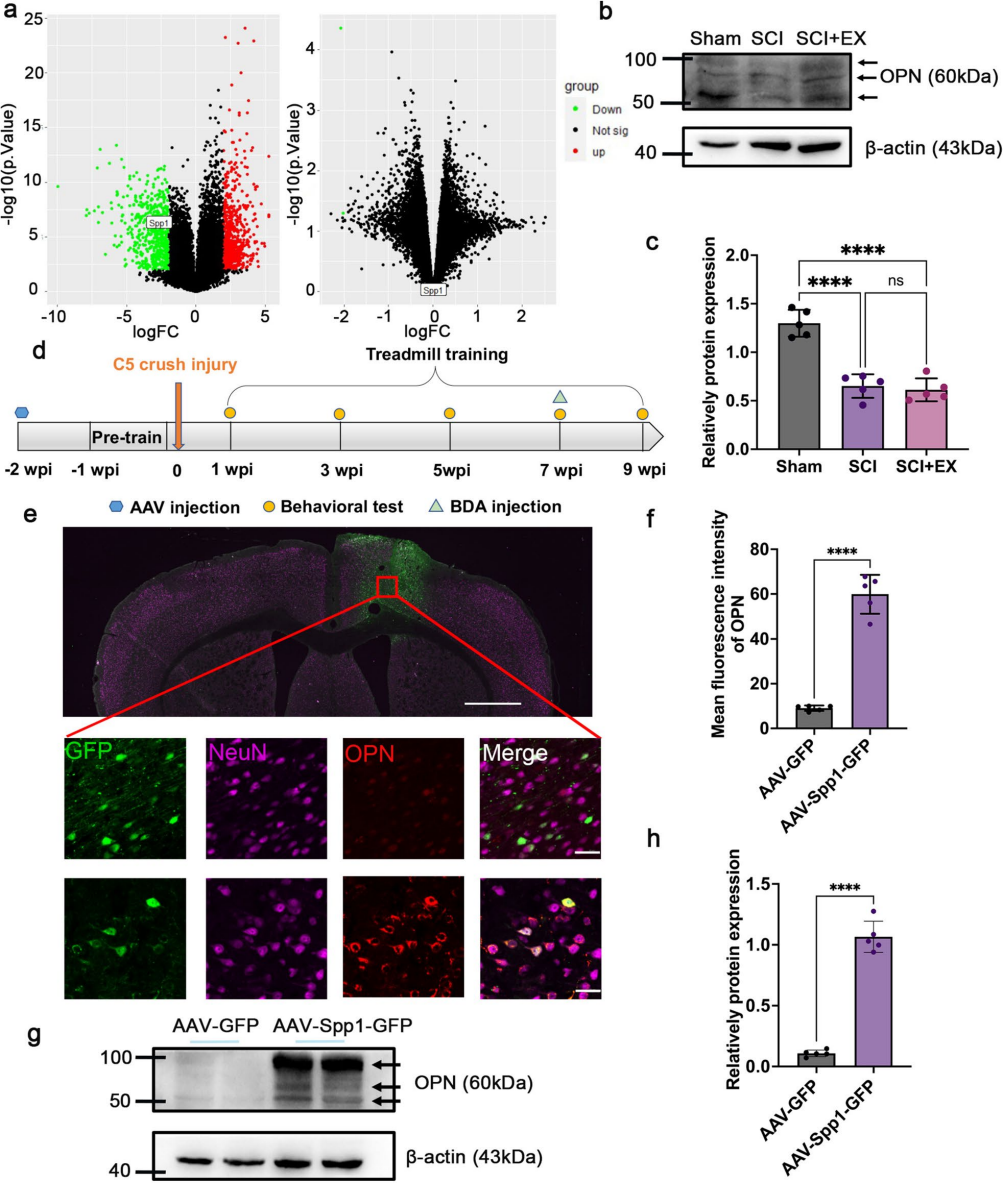
Overexpression of OPN at layer 5 of the right sensorimotor cortex. **a** The differentially expressed genes. Left: Sham vs. SCI. Right: SCI vs. SCI + EX. **b** The western blot results of the Sham (*n* = 5), SCI (*n* = 5), and SCI + EX (*n* = 5) groups showing the expression of OPN. **c** Quantitative analysis of (**b**). **d** Illustration of the experimental II timeline. **e** Representative fluorescence images of transverse brain sections from the AAV-Spp1-GFP (*n* = 5) and AAV-GFP (*n* = 5) groups showing GFP (green), NeuN (magenta), and OPN (red). Bar = 20 μm. **f** Quantification of OPN mean fluorescence intensity. **g** The western blot results of the AAV-Spp1-GFP and AAV-GFP groups showing the expression of OPN. **h** Quantitative analysis of (**g**)

Recent studies reported that co-expression of IGF-1/BDNF and OPN induces regrowth of retinal axons. The underlying mechanism could be that OPN sensitizes neurons’ responses to IGF-1 and BDNF after injury [35]. Further investigations have suggested that a combination of OPN and IGF-1 promotes CST regeneration [25, 27]. Considering the effect of treadmill training in promoting the expression of neurotrophins, we asked whether OPN plays a role in exercise-induced repairment after SCI. Bioinformatics analysis was used in this study, and the results suggested that secreted phosphoprotein 1 (SPP1) was significantly downregulated after SCI in rats. However, the Spp1 expression level was not changed after treadmill training in SCI model (Fig. 3a). Furthermore, Western blot results confirmed the downregulation of OPN after SCI, and there was no significant difference between the SCI and SCI + EX groups (Sham vs. SCI, *p* = 0.0002; SCI vs. SCI + ex *p* = 0.8322) (Fig. 3b-c). Given the potential roles of OPN in SCI and the activation effect of treadmill training on mTOR signaling, we overexpressed OPN in the sensorimotor cortex by injection of AAV-Spp1-GFP. The experimental timeline is shown in Fig. 3d.

Immunofluorescence staining showed successful expression of AAV at layer 5 of the right sensorimotor cortex (Fig. 3e). Then, the overexpression effect of AAV-Spp1-GFP was confirmed. Both immunofluorescence staining and Western blotting demonstrated that OPN expression was significantly upregulated in the AAV-Spp1-GFP-injected group (Fig. 3f-h).

### Combining OPN and treadmill training amplified the activation of mTOR in the sensorimotor cortex

**Fig. 4:**
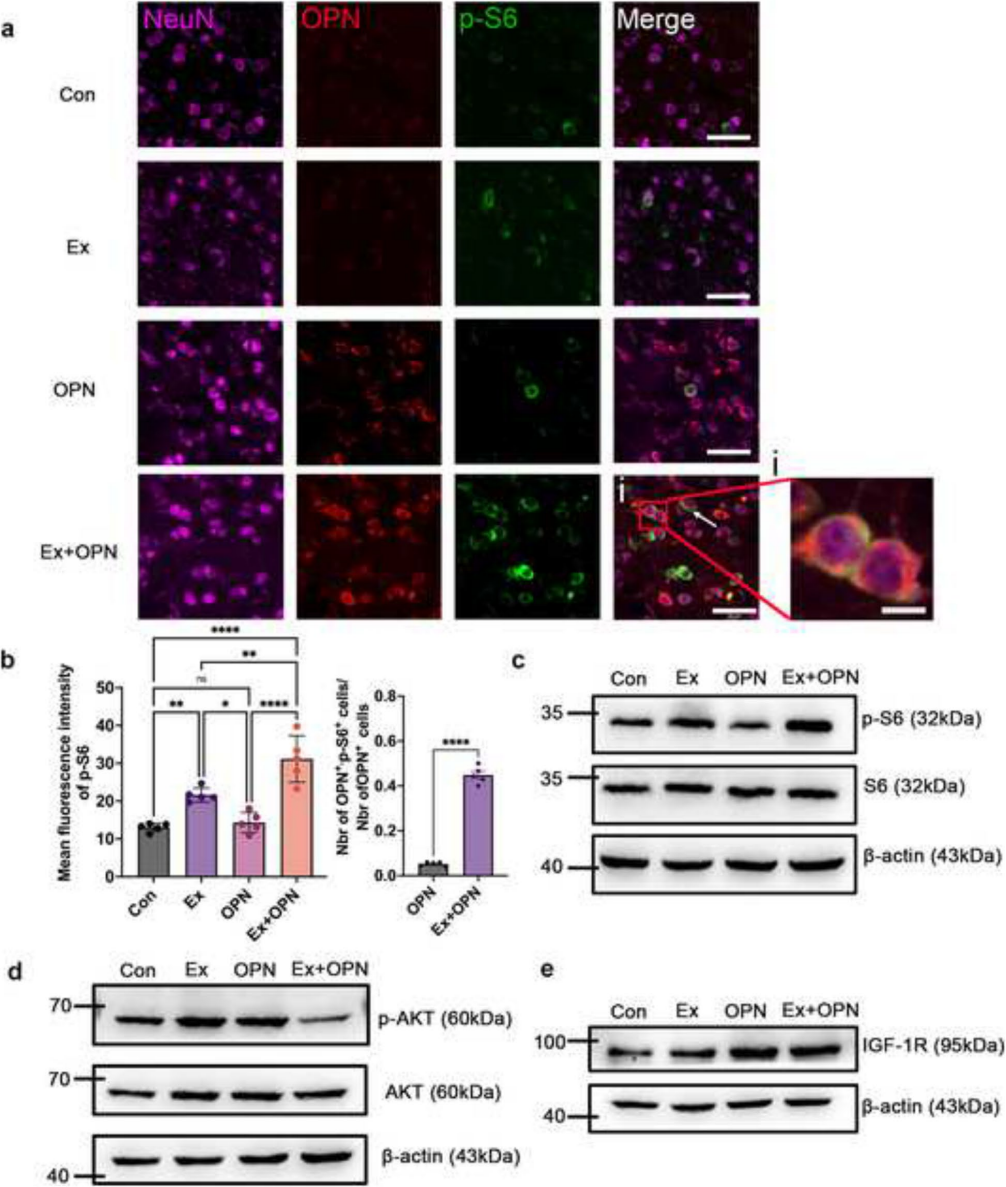
Amplified activation of mTOR by combining OPN and treadmill training. **a** Representative fluorescence images of transverse brain sections from the Con (*n* = 5), Ex (*n* = 5), OPN (*n* = 5), and Ex + OPN (*n* = 5) groups showing NeuN (magenta), OPN (red), and p-S6 (green). Bar = 25 μm. The white arrows indicate OPN^+^ and p-S6^+^ cells. **i** magnified images of a. **b** Quantification of the p-S6 mean fluorescence intensity (left) and the ratio of OPN^+^ and p-S6^+^ cells to total OPN^+^ cells. **c** The western blot results of the Con (*n* = 5), Ex (*n* = 5), OPN (*n* = 5), and Ex + OPN (*n* = 5) groups showing the expression of p-S6. **d** The western blot results of the Con, Ex, OPN, and Ex + OPN groups showing the expression of p-AKT. **e** The western blot results of the Con, Ex, OPN, and Ex + OPN groups showing the expression of IGF-1 receptor

Next, we investigated the effect of the combination strategy of OPN and treadmill training on the activation of mTOR signaling. Consistent with the results mentioned before, treadmill training significantly increased the phosphorylation level of S6 in the sensorimotor cortex. And there was no significant difference in the phosphorylation level of S6 between the Con and OPN groups. Interestingly, the expression of p-S6 was significantly increased in the Ex + OPN group compared with the Con and Ex groups (Ex + OPN vs. Con, *p* < 0.0001; Ex + OPN vs. Ex, *p* = 0.0008) (Fig. 4a-b). Furthermore, the OPN-positive cells were mostly p-S6-positive in the Ex + OPN group, which suggested the amplification effect of OPN in activating mTOR signaling (Fig. 4b). The contralateral side of the sensorimotor cortex showed a similar mTOR signaling activation level between the Ex and Ex + OPN groups. (Fig S3c-d). In addition, similar results were observed by Western blotting. The phosphorylation level of S6 in the Ex + OPN group was significantly higher compared with the Ex group (Fig. 4c, Fig S3e). Nevertheless, the level of AKT phosphorylation was lower in the Ex + OPN group than in the Con, Ex, and OPN groups. There was no significant difference among the Con, Ex, and OPN groups (Ex + OPN vs. Con, *p* < 0.0001; Ex + OPN vs. Ex, *p* < 0.0001; Ex + OPN vs. OPN, *p* = 0.0003) (Fig. 4d, Fig. S3f). AKT is upstream of mTOR signaling and could be negatively feedbacked by the level of S6 phosphorylation. Furthermore, IGF-1 receptor was upregulated in the OPN and Ex + OPN groups compared to the Con and Ex groups, respectively. There was no significant difference between the OPN and Ex + OPN groups. The expression of IGF-1 receptor was not enhanced by treadmill training (Ex + OPN vs. Con, *p* = 0.0021; Ex + OPN vs. Ex, *p* = 0.0207) (Fig. 4e, Fig. S3g). Collectively, OPN promoted the expression of the IGF-1 receptor, thus amplifying the effect of treadmill training on the activation of mTOR signaling.

### Combining OPN and treadmill training facilitated CST axon regeneration to a limited extent

**Fig. 5:**
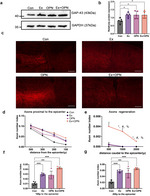
Combining OPN and treadmill training resulted in limited axonal regeneration and attenuated axonal dieback. **a** The western blot results of the Con, (*n* = 5), Ex (*n* = 5), OPN (*n* = 5), and Ex + OPN (*n* = 5) groups showing the expression of GAP-43. **b** The quantitative analysis of (**a**). **c** Representative fluorescence images of C5 segment spinal cord sagittal sections for the Con (*n* = 5), Ex (*n* = 5), OPN (*n* = 5), and Ex + OPN (*n* = 5) groups. The white arrows indicate regenerative axons. **d** Quantification of BDA-labeled CST axons in the spinal cord proximal to the lesion sites of the Con, Ex, OPN, and Ex + OPN groups. **e** Quantification of BDA-labeled CST axons in the spinal cord distal to the lesion sites of the Con, Ex, OPN, and Ex + OPN groups. **f**-**g** Quantitative analysis of (**d**) at different distances rostral to the lesion site. *, *p* < 0.05, Con vs. Ex + OPN. #, *p* < 0.05, Ex vs. Ex + OPN. %, *p* < 0.05, OPN vs. Ex + OPN. Nonparametric tests were used when the data did not conform to Gaussian distributions

Because combining OPN and treadmill training amplified the activation of mTOR in the sensorimotor cortex and mTOR signaling was one of the several important regulators to control CST regeneration, we next explored whether combining OPN and treadmill training facilitated CST axon regeneration. First, we determined the change in growth-associated protein-43 (GAP-43) expression level, which is related to axonal growth and regeneration [36]. The expression level of GAP-43 was improved in the Ex + OPN group compared with the Con group (Ex + OPN vs. Con, *p* = 0.0121) (Fig. 5a-b). Then, the situation of CST axon regeneration and dieback was investigated. As shown in Fig. 5C, BDA^+^ CST fibers were present in the white matter in an orderly manner. A limited portion of fibers within the gray matter was noted to regenerate and innervate the spinal cord caudal to the lesion in the Ex + OPN group (Fig. 5c).

The axon number index of BDA^+^ CST fibers at different distances rostral and caudal to the lesion site was also quantified (Fig. 5d-g). At 200 μm and 100 μm rostral to the lesion site, the axon number index of BDA + CST fibers was significantly increased in the Ex, OPN, and Ex + OPN groups (Fig. 5f, g). More importantly, an increase in the axon number index was found only in the Ex + OPN groups 500–2000 μm caudal to the lesion site (Fig. 5e). This increase between the Ex + OPN group and the Con group was statistically significant. Figure S4 presented the regenerated axons 2000 μm caudal to the lesion site. Together, combining OPN and treadmill training attenuated CST axon dieback and facilitated limited CST axonal regeneration.

### Functional recovery after treadmill training combined with OPN

**Fig. 6:**
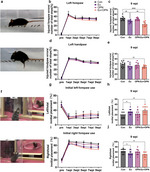
Behavioral test outcomes. **a** Representative photograph of the horizontal ladder task. **b** The error percent of the injured forelimb in the horizontal ladder task. **c** Quantification of the error percent of the injured forelimb at 9 wpi. **d** The error percent of the injured hindlimb in the horizontal ladder task. **e** Quantification of the error percent of injured hindlimb at 9 wpi. **f** A representative photograph of the rearing test. **g** The initial usage percent of the left forepaw in the rearing test. **h** Quantification of the initial usage percent of the left forepaw at 9 wpi. **i** The initial usage percent of the right (uninjured) forepaw in the rearing test. **j** Quantification of the initial usage percent of the right forepaw at 9 wpi

Finally, we performed behavioral tests to define functional recovery. The denervated forelimbs showed severe locomotor deficits during the first week after the injury as mice crossed the horizontal ladder. The error percentage for the injured forelimb showed a significant increase in all groups at 1week post injury (wpi), followed by a gradual decrease in the subsequent weeks after SCI (Fig. 6a-b). However, mice in the EX + OPN group showed a moderately lower error percentage of the injured forelimb than those in the Con and OPN groups at 9 wpi (Fig. 6c). In addition, the error percentage of the injured forelimb was lower in the Ex + OPN group than in the Ex group with statistical difference. The error percentage of the hindlimbs increased at 1 wpi in all groups and exhibited a gradual decrease over time (Fig. 6d). There was no significant difference among the four groups at any time point, although the Ex + OPN groups showed a lower error percentage compared with the Con group at 9wpi (Fig. 6e).

In the rearing test, there was a significant decrease in the initial use of the left (injured) forepaw, accompanied by a substantial increase in the initial use of the right forepaw following the injury (Fig. 6f-g, i). After 8 weeks of treadmill training, the usage of the left forepaw in the Ex group was slightly improved compared to that in the Con group without statistical difference. The combined strategy minorly increased the usage of the left forepaw and statistically significant difference was found between the Ex + OPN group and the Con group (Fig. 6g-h). The same pattern was observed in the initial usage of the right forepaw, too (Fig. 6i-j).

Behavioral test outcomes indicated that treadmill training facilitated a modest improvement in motor function in the injured limbs, and this effect was further enhanced by OPN.

## Discussion

The current study showed the effect of treadmill training on promoting neurotrophins (BDNF, IGF-1) expression and activating mTOR signaling in a C5 crush spinal cord injury model. Our data also showed that combining treadmill training and OPN strongly upregulated p-S6 and promoted a limited portion of CST axons to regenerate to the spinal cord caudal to the lesion.

The effects of exercise training are multifaceted and intricate, impacting various aspects of the central nervous system. As a main clinical strategy in rehabilitation medicine, it has been widely proven that exercise training positively affects the circadian rhythm, central metabolism, cardiovascular function, and stress responses of the CNS [37–40]. However, the benefit of exercise training may relate to specific forms and training intensity. Recent research by Chen and colleagues revealed that treadmill training, conducted at a speed of 12 m/min, resulted in the strengthening of postsynaptic densities (upregulation of PSD-95 and SNAP25), enhanced synaptic transmission, increased neuron activity, and boosted axonal myelination (elevated myelin basic protein intensity) through the activation of mTOR signaling in layer 5, as indicated by increased phosphorylation of ribosomal protein S6 [41]. Another study by Côté et al. demonstrated that treadmill training promoted the expression of neurotrophins (BDNF, NT-3, NT-4) in a T12 complete spinal transection model [42]. This finding indicates that treadmill training plays a potential role in CNS repair and plasticity. On the other hand, several studies confirmed that the activation of mTOR signaling in CNS neurons could prompt axonal regeneration by knocking out PTEN or manipulating other genes in spinal cord injury or stroke models [25, 43, 44]. Therefore, the utilization of treadmill training for mTOR activation holds promise for clinical application. However, our data did not prove that treadmill training alone could promote CST axon regeneration, although the expression of BDNF and IGF-1 was upregulated and the phosphorylation of ribosomal protein S6 after injury. Unfortunately, neurotrophins exhibit limited efficacy in protecting neurons in disease models [45]. For instance, it was observed that BDNF demonstrated a substantial capacity for promoting the regeneration of rubrospinal tract axons when administered immediately after injury. However, its impact appeared limited when administered at the two-month post-injury mark [23, 46]. This possibly due to the downregulation of neurotrophins receptors, such as IGF-1 receptors α and β [23, 24]. A recent study reported that OPN can sensitize neurons to the effects of neurotrophins and enhance axon regeneration in retinal ganglion cells. OPN was found to increase IGF-1 receptor expression and phosphorylation of IGF-1 receptor in a T10 lateral hemisection model [25, 35]. Similarly, our data showed that combining treadmill training and OPN induced better functional recovery compared to treadmill training alone and slight CST axon regeneration, which was not observed in other groups. Given that OPN is a soluble protein, soluble protein therapeutics hold great promise for treating various neural injuries and neurological diseases. This suggests that combining treadmill training with OPN presents a promising new strategy for spinal cord injury repair.

OPN is a multifunctional protein found in various cells and tissues [26]. Numerous studies have highlighted its involvement in pro-inflammatory processes and tissue repair [47–50]. OPN has been shown to enhance NK cell migration and activate NK cells [51]. Moreover, Fu and his colleagues have reported that OPN expression was observed in microglia and macrophage following spinal root avulsion [52]. To our knowledge, inflammation gradually escalates in the initial days following spinal cord injury [53]. As a result, the expression of OPN is upregulated in this phase as confirmed by Hashimoto et al. [54]. However, as spinal cord injury progresses, inflammation tends to subside. Additionally, the inhibitory microenvironment in the chronic phase of SCI makes neural repair more challenging to achieve [55]. Consequently, it’s reasonable to infer that OPN expression decreases during the chronic phase of SCI. In a spinal cord injury model, grape seed extract (GSE) was found to have contrasting effects on OPN expression [56]. GSE was shown to reduce the expression of interleukin (IL)-4, IL-13, and vascular endothelial growth factor (VEGF), thus ameliorate inflammatory response [57]. In addition, OPN could be upregulated by activating phosphoinositide 3-kinase (PI3K), extracellular signal-regulated kinase (ERK), and c-Jun NH2-terminal kinase (JNK) in LPS- stimulated macrophages [50]. Therefore, the regulatory signals governing the expression of OPN are intricate.

Promoting axonal regrowth stands as a pivotal element in the realm of SCI treatment approaches [55]. Over the past several decades, a large stride has been made in promotion of axonal regeneration in SCI. The regenerative potential of axons is influenced by a multitude of factors which can be broadly categorized into intrinsic and extrinsic factors. To activate the intrinsic growth potential of injured neurons, Liu et al. deleted Pten in a T8 dorsal hemisection SCI model and found the robust regeneration of CST tract [43]. Extrinsic factors, such as chondroitin sulfate proteoglycans (CSPGs), can directly or indirectly inhibit axonal repair. Chondroitinase ABC (ChABC) is an enzyme that degrades CSPGs [58]. Bradbury and his colleagues observed regeneration of both sensory projections and corticospinal tract axons when ChABC was administered immediately after a C4 dorsal column crush injury [59]. In addition, neurotrophins play a role in axon repairment. Jin et al. reported that the application of BDNF to the injured spinal cord promoted the regeneration of the rubrospinal tract, reticulospinal tract, and vestibulospinal tract in a C3 complete unilateral hemisection injury model [60]. Liu and her team found that IGF-1 treatment significantly facilitated CST axonal sprouting across the midline compared to the control group [25]. The transplantation of all kinds of stem cells such as neural stem cells has been explored as a promising therapy for axonal regeneration. Lu and his team noted significant axonal growth originating from the grafts, extending over considerable distances and establishing synaptic connections within the T3 complete transection spinal cord rats that received human neural stem cell transplants [61]. Rehabilitation has also been reported to promoting axonal repairment in SCI [6]. Moderate intensity treadmill training has been confirmed to enhance CST axonal sprouting and functional recovery by activating mTOR signaling in an incomplete C5 crush SCI model [62]. Now, researchers may combine several potential therapies to further enhance neural regeneration and functional recovery.

It should be noted that combining treadmill training with OPN resulted in axonal regeneration, albeit to a limited extent (axon index ~ 0.003). In studies led by Zhigang He, they observed robustly regenerated axons (fiber number index ~ 0.023, approximately 7–8 times greater than our data) following PTEN deletion in the T8 dorsal hemisection model [43]. Several factors might be able to explain this. First, it is unknown whether eight weeks was sufficient, as axonal regeneration takes considerable time. In previous experiments where IGF-1 and OPN were overexpressed via virus injection into the cortex, it took 12 weeks to observe axonal regeneration in a T10 lateral hemisection model [25]. Axonal regeneration occurred 8 weeks after T8 dorsal hemisection in PTEN deletion mice with a regenerated fiber number index of approximately 0.011, while the regenerated fiber number index was approximately 0.023 at 12 weeks after T8 complete crush in Pten deletion mice [43]. Therefore, a prolonged training period might be necessary to induce robust axonal regeneration. Second, it is difficult to confirm the extent to which treadmill training promotes the expression of neurotrophins (e.g., BDNF and IGF-1). In previous experiments where a high concentration of BDNF (20,000 ng/µl) was directly administered to the spinal cord using a small pledget of gelfoam soaked in BDNF four times within an hour, researchers did not observe rubrospinal tract axonal regeneration in cervical axotomy mice [23]. However, when a mixed solution of 0.5 µl AAV (OPN: 1 × 10^13, IGF: 5 × 10^12, CNTF: 5 × 10^12 gc/ml) was injected into the spinal cord and delivered fibroblast growth factor 2 (FGF), epidermal growth factor (EGF), and glial-derived growth factor (GDNF) via biomaterial depots of synthetic hydrogels in mice and rats with severe crush SCI, Anderson and his colleagues observed very robust axonal regeneration [63]. Hence, it’s possible that the amount of neurotrophins induced by treadmill training may not have been sufficient for axonal regeneration in our study. Lastly, it’s worth noting that BDA may not label all the targeted axons comprehensively. Robust CST axonal regeneration was observed in crym-GFP transgenic mice rather than BDA tracing mice when *ngr1* was knocked out. This comprehensive CST labeling with the mu-crystallin transgene was found to be 10 times more efficient than using BDA [64]. Therefore, it’s possible that some regenerated axons may have been missed in our study.

In the current study, although axonal regeneration in the Ex + OPN group was limited, behavioral data showed that mice in the combined group acquired functional recovery. Treadmill training activates mTOR pathways of the whole brain, indicated by upregulating the expression of p-S6. Both sprouted and regenerated CST axons could lead to behavioral functional recovery in SCI mice [25].This possibly promoted CST axonal sprouting on both sides of the spinal cord. Lee and his team discovered that the activation of mTOR signaling through PTEN deletion resulted in a substantial increase in trans-midline sprouting of CST axons from the intact side into the denervated side in unilateral pyramidotomy transgenic mice [65]. Additionally, chronic motor cortex electrical stimulation was found to activate mTOR signaling and thus promote ipsilateral CST axon growth [66]. Therefore, we propose that the neural substrates that promote functional recovery (exercising or exercising + OPN) include midline-crossing sprouted axons from the spared dorsolateral CST tract. However, these axons may contribute to some effects, not all. Based on our data, combining treadmill training with OPN strongly activated mTOR signaling in the right sensorimotor cortex. Additionally, our previous studies reported that treadmill training promotes CST axon sprouting, close connectivity of sprouted axons with neurons, and the reforming of synaptic connectivity [62, 67]. Therefore, the sprouting and regeneration of the left side CST (injured) axons may be another reason that promotes functional recovery. Many studies have demonstrated that exercise training plays multiple roles in promoting functional recovery after SCI. For instance, exercise serves as a dependable catalyst for activity-dependent neuroplasticity and synaptic plasticity, both of which play a pivotal role in facilitating functional recovery following SCI [6]. Goldshmit and colleagues have recently illustrated that treadmill training at a speed of 12 m/min prompted the sprouting of axons that establish synaptic connections with neurons in the gray matter proximal to the lesion site. This regimen also led to improvements in behavioral performance, as evidenced by enhanced BBB scores, grid walking, and climbing abilities, in a murine model of T10 hemisection [68]. We also observed that moderate-intensity treadmill exercise stimulated the expression of mTOR-dependent motor cortical neurotrophic factors and led to enhanced functional recovery in a cervical crush injury model [62]. On the other hand, treadmill training can promote myelin repair, which induces functional recovery after SCI. Jensen et al. reported that voluntary exercise increased the generation of new oligodendrocytes, promoted the expression of myelin basic protein, and increased remyelinated axon density (2.11-fold) in a lysolecithin model of toxin-induced demyelination [69–71]. Finally, several studies have indicated that the local inflammatory response within the spinal cord following SCI plays a significant role in secondary damage, exacerbating functional impairments. Exercise presents itself as a non-pharmacological approach to mitigate these inflammatory responses by reducing the expression of inflammatory mediators, such as IL-1β [72–74].

There are limitations in our study. On the one hand, we traced only one side of the CST tract, which means that the axonal sprouting of spared dorsolateral CST could not be analyzed. Considering the recovery of the injured limbs and the limited regeneration of injured CST axons, spared dorsolateral CST axons deserve to be examined. On the other hand, the analysis of mTOR activation in cortical neurons was not specific enough, thus weakening the relationship of CST axon regeneration with the activation of mTOR signaling induced by treadmill training plus OPN. For example, a persistent neuronal retrograde tracer could be employed to specifically label pyramidal neurons. Then, one can observe the activation of mTOR signaling in those neurons. The mini ruby used in our study had a limited duration, fading after just four weeks [75]. New evidence has shown that AAV-HiRet-GFP injection would be a more suitable choice [25].

In conclusion, our data demonstrated that eight weeks of treadmill training combined with AAV-OPN injection into the cortex could activate the mTOR signaling pathway, promote mild CST axonal regeneration, and improve functional performance. This work provides a new insight and clinical translatable possibilities for exploring CNS regeneration.

## Materials and methods

### Animals and groups

Adult female C57B/6 mice, with body weights falling within the range of 18 to 20 g, were utilized as the subjects for this investigation. Mice were accommodated in an animal housing facility under a 12:12 h light-dark cycle, with water and food available ad libitum. Ethical clearance was duly obtained from the Animal Experiment Ethics Committee of Chongqing Medical University.

The study comprised two experiments. In Experiment I, the mice were randomly assigned to three groups: Sham group (Sham, *n* = 10), spinal cord injury group (SCI, *n* = 10), and spinal cord injury + exercise group (SCI + Ex, *n* = 10). Experiment II involved the random allocation of mice into four groups: the control group (Con, *n* = 10), the exercise group (Ex, *n* = 10), the OPN group (OPN, *n* = 10), and the exercise + OPN group (Ex + OPN, *n* = 10).

#### Virus injection and anterograde tracing of corticospinal tract

AAV9-Spp1-GFP(64012-1), negative control virus (CON336, AAV-GFP) and biotinylated dextran amine (BDA) were injected into the right sensorimotor cortex of the mice, as previously described [20]. AAV9 was injected 2 weeks before the injury, while BDA was injected 7 weeks after the injury.

After inducing anesthesia with intraperitoneal injection of 0.5% sodium pentobarbital (50 mg/kg; Shanghai Pharmaceutical Factory), the skin overlying the skull was shaved and sterilized. Subsequently, the right sensorimotor cortex was exposed by means of a dental drill. A total volume of 2.4 µl (0.6 µl per site at 4 different sites, administered at a rate of 0.2 µl/min) containing AAV9 or BDA was stereotaxically injected into the layer 5 sensorimotor cortex on the right side (coordinates: bregma − 0.4, -0.6 mm; lateral 0.8, 1.2 mm; depth 0.7 ~ 0.9 mm). This procedure was carried out using a Hamilton syringe attached to a glass-pulled pipette. After injection, the syringe was left in place for 3 min. Finally, the skin was sutured and disinfected.

### Incomplete C5 crush injury

Mice were initially weighed and subsequently anesthetized via intraperitoneal injection of 0.5% sodium pentobarbital (50 mg/kg). The neck area’s skin was prepared through shaving and disinfection. To expose the fifth cervical spinal cord segment, a laminectomy was performed, and the dorsal root entry zones were penetrated with a 26-gauge needle. Specifically, modified Dumont #5 forceps with a width of approximately 200 μm and a length of around 2 mm, featuring a marker placed at a 1 mm distance from the tip, were meticulously employed. Both prongs of these finely tapered forceps were inserted into the bilateral dorsal horn gray matter to a depth of approximately 1 mm [76]. The forceps were then closed and held for 15 s before being withdrawn and reinserted to repeat the compression. Subsequently, one prong of another finely tapered forceps was inserted into the left dorsal horn gray matter to a depth of around 0.8 mm, while the other prong remained outside the spinal cord [76]. The forceps were again closed and held for 15 s, and the process was repeated. Following the injury, the muscles and skin were sutured to complete the procedure. It’s worth noting that the right dorsolateral funiculus, encompassing the right rubrospinal tract and the right lateral corticospinal tract, remained intact, thereby preserving some motor function on the right side.

### Retrograde tracing

A 5% solution of Mini Ruby, an anterograde tracer (Thermo, USA), was utilized to inject and retrogradely label neurons within the sensorimotor cortex. The Mini Ruby solution (0.2 µl per site × 4 sites, administered at a rate of 0.05 µl/min) was injected via a Hamilton syringe connected to a glass-pulled pipette. This injection procedure took place while the mice were under anesthesia. The specific injection coordinates were established on both the left and right sides (lateral: 1.1 mm; depth: 0.5 mm) of the spinal cord midline, situated between the C6 and C7 levels.

### Treadmill training

Before the injury, a preliminary treadmill training regimen was administered to all mice over a span of 3 days, involving a speed of 9 m/min for a duration of 20 min. The assessment of their maximum running speed occurred on the day prior to the injury. This evaluation encompassed a warmup period at 9 m/min for 5 min, followed by a gradual augmentation in speed employing specific acceleration rates (4.5 m/min^2). The mice were deemed to have reached their maximal running speed upon receiving a sequence of shocks. To track alterations in the mice’s physical condition, the maximum running speed evaluation was conducted every two weeks.

Mice within the Ex group and the EX + OPN group were exposed to eight weeks of treadmill training, carried out five times per week for a duration of 30 min each session. Commencing one-week post-injury, mice underwent training sessions at 50% of their maximum running speed. For this purpose, a 6-lane treadmill (Nanjing Calvin Biotechnology Co., LTD, China) with an initial speed of 9 m/min was employed.

### Immunofluorescence staining

On the day following the final behavioral test, all mice were euthanized. Anesthesia was induced using 0.5% sodium pentobarbital (50 mg/kg), followed by perfusion of cold PBS through the heart, and subsequently, 4% paraformaldehyde (PFA) perfusion. The brains and spinal cords were meticulously isolated and then post-fixed overnight in 4% PFA. Subsequently, a gradual dehydration process was carried out using 0.1 M phosphate buffer containing increasing concentrations of sucrose (18%, 24%, 30%) over a span of 3 days. After carefully blotting the surface of the liquid, the brains and spinal cords were embedded in Tissue-Tek (Sakura). Longitudinal sections of the spinal cords, measuring 20 μm in thickness, and transverse sections of the brains, also measuring 20 μm in thickness, were obtained. All these tissue sections were carefully categorized and stored at -80 °C.

For immunostaining, the cryosections of the brains and spinal cords were allowed to thaw at room temperature for 30 min, followed by three 5-minute washes in PBS. Thereafter, the sections were incubated with 0.03% Triton X-100 for 30 min, followed by three additional washes in PBS (each lasting 5 min). Subsequently, the sections were subjected to a 60-minute incubation in Blocking Buffer for Immunol Staining. Antibodies against GFP (1:200, Abcam, USA), p-S6 (1:200, Cell Signaling, USA), NeuN (1:800, Millipore, USA), GFAP (1:2000, ThermoFisher, USA), and OPN (1:150, Biotechnology, USA) were employed. The corresponding primary antibodies were applied overnight at room temperature. On the subsequent day, after three 5-minute PBS washes, the sections were incubated with the appropriate secondary antibodies (anti-mouse, anti-goat, anti-rabbit, and anti-guinea pig antibodies conjugated to Alexa Fluor 488, 594, or 647 (1:400; Jackson Laboratories, USA)) for 60 min at room temperature. BDA-traced CST axons were visualized using streptavidin labeled with Alexa Fluor 594 (1:400, Thermo Fisher, USA). After three additional three PBS washes (each lasting 5 min), the sections were counterstained with DAPI (10 µg/mL, Sigma, USA) for 2 min, followed by three more rounds of 5-minute PBS washes. At last, sections were sealed in Antifade Polyvinylpyrrolidone Mounting Medium.

### Western blot

To prepare cortical lysates, mice were flushed with an ample amount of cold PBS. The whole brain was isolated, and the sensorimotor cortex tissue was rapidly excised using a sterile blade before being stored at -80 °C. For spinal cord lysates, a segment measuring 0.5 mm in length caudal and rostral to the epicenter was isolated. The tissues were homogenized in RIPA Lysis Buffer (Beyotime, China) containing protease inhibitors (Beyotime, China) and phosphatase inhibitors (Beyotime, China). An ultrasonic cell crusher set at 20% power was used for homogenization for 3 min. The lysates were subsequently clarified through centrifugation using a tabletop centrifuge (Dynamica, Japan) at 16,000 × g for 20 min at 4 °C. Following centrifugation, the supernatant was collected, and the protein concentration was gauged using a BCA protein assay (ThermoFisher scientific, USA). Finally, the SDS–PAGE sample loading buffer (6X, Beyotime, China) was added, followed by boiling in water for 10 min. After cooling, the proteins were stored at -80 °C.

Protein separation was carried out using SDS/PAGE (10%) following established protocols. Subsequently, the proteins were transferred to nitrocellulose membranes (0.22 μm; Absin, China). Blots were blocked for 90 min in a solution containing 5% nonfat powdered milk in Tris-buffered saline with 0.1% Tween-20 (TBST, Beyotime, China), or for 30 min using QuickBlock™ Blocking Buffer (Beyotime, China) at room temperature. The membranes were then incubated overnight at 4 °C with antibodies targeting the following: total S6 (1:1000; Cell Signaling, USA), pS6 (1:1000; Cell Signaling, USA), pAKT (1:1000; Cell Signaling, USA), total AKT (1:1000; Cell Signaling, USA), BDNF (1:1000; Cell Signaling, USA), IGF-1 (1:1000; Cell Signaling, USA), OPN (1:2000; Biotechnology, USA), GAP-43 (1:1000; Boster, China), β-actin (1:5000; Proteintech, China), and GAPDH (1:1000; Cell Signaling, USA). All primary antibodies were diluted using primary antibody dilution buffer (Beyotime, China). Secondary antibodies, including anti-rabbit, anti-mouse, or anti-goat IgG conjugated to HRP (1:3000, 1:5000, and 1:3000, respectively; Servicebio, China), were used. The antigen-antibody complexes were visualized using BeyoECL star (Beyotime, China) and captured with a Gel Imager System (Tanon, China). ImageJ (National Institutes of Health, Bethesda, USA) was utilized to measure the integrated gray values of each band.

### Identification of differentially expressed genes (DEGs)

GEO2R (http://www.ncbi.nlm.nih.gov/geo/geo2r) is an interactive web tool that facilitates the comparison of two or more groups of samples within a GEO series to identify genes exhibiting differential expression under various experimental conditions. In order to obtain differentially expressed genes, the samples of GSE52763 and GSE45006 were respectively selected to compare [77, 78]. GEO2R was used to compare samples of GSE52763 and GSE45006. Subsequently, we downloaded the list of differentially expressed genes, applying criteria of a *P* value (*p*) < 0.05 and |log2-fold change (FC)| > 2. R (version 4.0.5) was utilized for visualizing these DEGs, and the results are presented in the form of volcano plots.

### Axon regeneration

Longitudinal sections through the lesion were subjected to staining for BDA and GFAP to delineate lesion edges. To provide an approximate quantification of the total axon count, we assessed the number of axons at the medulla level. Specifically, a series of vertical lines was drawn at 100 μm intervals along the BDA-labeled axons rostral to the lesion site. We then tallied the axons intersecting these vertical lines. A similar approach was employed caudal to the lesion site to quantify individual axons, with vertical lines drawn at 500, 1000, and 2000 μm from the lesion site, respectively. The results, expressed as an axon number index, were normalized relative to the total axon count.

### Behavioral tests

All behavioral analyses were conducted by personnel blinded to the group inclusion.

### Irregular horizontal ladder

The assessment of error percentage, encompassing slip, miss, or drag, for both the left forelimb and hindlimb, was conducted using an irregular horizontal ladder. This horizontal ladder employed possessed dimensions of 5.2 cm width and 30 cm height, featuring 31 rungs spaced at intervals of 1.3 cm. To eliminate any learned behaviors, five random rungs were intentionally excluded from the design of the 31-rung ladder. During evaluation, each mouse traversed the ladder five times per time point, with mid-pathway reversals being disregarded. A high-definition camera (SONY HDR-CX610) was utilized to record five instances of each mouse traversing the ladder, following which the total steps and error steps of the left limb during these five movements were quantified [76]. To calculate the limb-specific error percentage, we normalized the count of error steps by the total step count.

### Cylinder rearing test

Forelimb functional recovery was assessed by calculating the proportion of injured forelimbs capable of supporting their own weight against a wall Mice were placed inside a 20 cm high and 15 cm diameter plexiglass cylinder for a 15-minute observation period during which their forelimb movements were carefully monitored. To ensure comprehensive observation of forepaw motion regardless of the mouse’s orientation, two mirrors were strategically positioned behind the cylinder at an angle [76]. The entire process was captured through video recording using an iPhone 6s (Apple, USA). A total of 10 rearing sessions were recorded to assess initial forepaw use. The initial placement was categorized as “left” or “right” when the corresponding forepaw independently supported the body’s weight for over 0.25 seconds. If both forepaws were engaged in self-support for over 0.25 seconds, the situation was classified as “both”. Ratios pertaining to the initial utilization of the left forepaw (left/total initial placements), the right forepaw (right/total initial placements), and both forepaws (both/total initial placements) were subsequently analyzed for each designated time point.

### Statistical analysis

All data are depicted as the mean ± standard error of the mean (SEM). Statistical analysis involved one-way ANOVA with Bonferroni’s post hoc analysis and unpaired Student’s t-test. In cases where data deviated from Gaussian distributions, nonparametric tests were applied. These statistical analyses were carried out using GraphPad Prism software (version 9.2), and statistical significance was defined at *p* < 0.05.

### Supplementary Information

**Additional file 1: Fig. S1.** Features of incomplete C5 crush. (a) Representative coronal spinal cord graphs of HE staining in the SCI and Sham group. Bar = 500 μm. (i) (ii) magnified images of the two groups. Bar =50 μm. (b) Representative fluorescence images of transverse brain sections from the Sham and SCI groups showing NeuN staining (green) and Mini ruby staining (red). Bar = 200 μm. (c) (d) Representative magnified images of the two groups. Bar =50 μm. (e) Quantification of the number of Mini ruby-positive cells in (c) and (d). (f) The BMS score of the Sham and SCI groups. Data are presented as the means ± SEMs. unpaired student test. *, *p* < 0.05; **, *p* < 0.005. **Fig. S2.** Epicenter area (a)Representative fluorescence images of longitudinal spinal cord sections from the Con, Ex, OPN, and Ex+OPN groups showing GFAP (green). (b) Quantification of the epicenter area. **Fig. S3.** (a) The quantitative analysis of BDNF. (b) Quantitative analysis of IGF-1. (c) Representative fluorescence images of transverse brain sections for the Con, Ex, OPN, and Ex+OPN groups showing NeuN (green) and p-S6 (red). Bar = 25 μm.(d) Quantification of the p-S6 mean fluorescence intensity. (e) Quantitative analysis of p-S6/S6. (f) Quantitative analysis of p-AKT/AKT. (g) Quantitative analysis of IR. **Fig. S4.** Long-distance regeneration in the Ex+OPN group. (a) Representative fluorescence images of longitudinal spinal cord sections from the Ex+OPN group. showing GFAP (green) and BDA (red). Bar = 500 μm. (b)(c) Magnification of the areas in the dotted box of A. Bar = 100 μm. (d) Magnification of the areas in the dotted box of c. Bar = 50 μm.

## Data Availability

Upon request, the corresponding author will provide the relevant data.
