# Supplementary material for: Osteopontin enhances the effect of treadmill training and promotes functional recovery after spinal cord injury
Source: Mol Biomed. 2023 Nov 28;4:44. doi: 10.1186/s43556-023-00154-y (PMC10684450; doi:10.1186/s43556-023-00154-y)
Supplement: Supplementary file 1 — Additional file 1: Fig. S1. Features of incomplete C5 crush. (a) Representative coronal spinal cord graphs of HE staining in the SCI and Sham group. Bar = 500 μm. (i) (ii) magnified images of the two groups. Bar =50 μm. (b) Representative fluorescence images of transverse brain sections from the Sham and SCI groups showing NeuN staining (green) and Mini ruby staining (red). Bar = 200 μm. (c) (d) Representative magnified images of the two groups. Bar =50 μm. (e) Quantification of the number of Mini ruby-positive cells in (c) and (d). (f) The BMS score of the Sham and SCI groups. Data are presented as the means ± SEMs. unpaired student test. *, p < 0.05; **, p < 0.005. Fig. S2. Epicenter area (a)Representative fluorescence images of longitudinal spinal cord sections from the Con, Ex, OPN, and Ex+OPN groups showing GFAP (green). (b) Quantification of the epicenter area. Fig. S3. (a) The quantitative analysis of BDNF. (b) Quantitative analysis of IGF-1. (c) Representative fluorescence images of transverse brain sections for the Con, Ex, OPN, and Ex+OPN groups showing NeuN (green) and p-S6 (red). Bar = 25 μm.(d) Quantification of the p-S6 mean fluorescence intensity. (e) Quantitative analysis of p-S6/S6. (f) Quantitative analysis of p-AKT/AKT. (g) Quantitative analysis of IR. Fig. S4. Long-distance regeneration in the Ex+OPN group. (a) Representative fluorescence images of longitudinal spinal cord sections from the Ex+OPN group. showing GFAP (green) and BDA (red). Bar = 500 μm. (b)(c) Magnification of the areas in the dotted box of A. Bar = 100 μm. (d) Magnification of the areas in the dotted box of c. Bar = 50 μm. [file 43556_2023_154_MOESM1_ESM.docx]

Supplementary Materials for

**Osteopontin enhances the effect of treadmill training and promotes functional recovery after** **spinal cord injury**

Yunhang Wang et al.

Corresponding authors: Botao Tan: [303518@cqmu.edu.cn](mailto:303518@cqmu.edu.cn);Ying Yin Email:[300735@cqmu.edu.cn](mailto:300735@cqmu.edu.cn)

The Word includes:

Supplementary figure Fig S1-S4

Supplementary figure 1


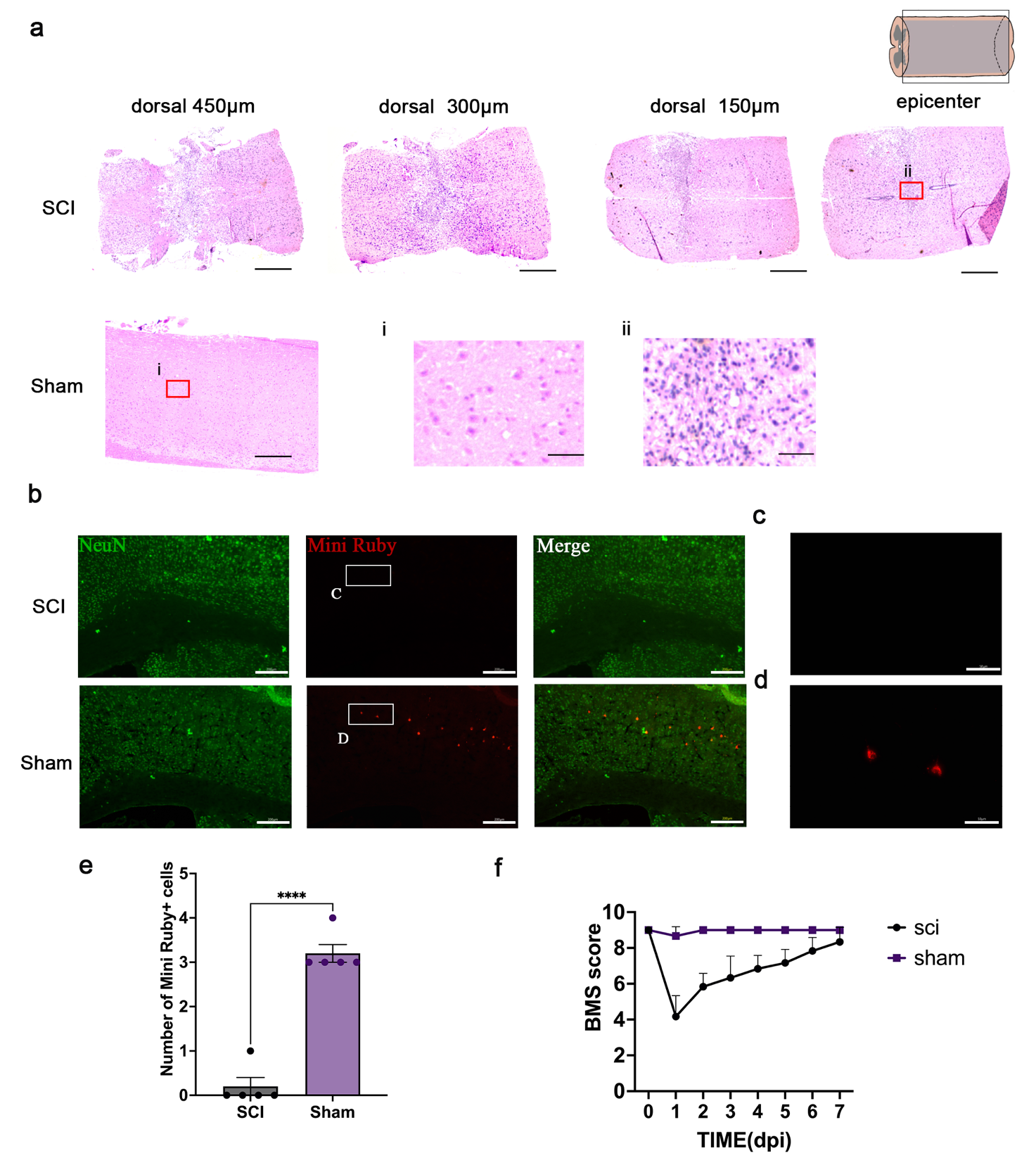


**Figure S1. Features of incomplete C5 crush.** (a) Representative coronal spinal cord graphs of HE staining in the SCI and Sham group. Bar = 500 μm. (i) (ii) magnified images of the two groups. Bar =50 μm. (b) Representative fluorescence images of transverse brain sections from the Sham and SCI groups showing NeuN staining (green) and Mini ruby staining (red). Bar = 200 μm. (c) (d) Representative magnified images of the two groups. Bar =50 μm. (e) Quantification of the number of Mini ruby-positive cells in (c) and (d). (f) The BMS score of the Sham and SCI groups. Data are presented as the means ± SEMs. unpaired student test. *, *p* < 0.05; **, *p* < 0.005.

Supplementary figure 2

**
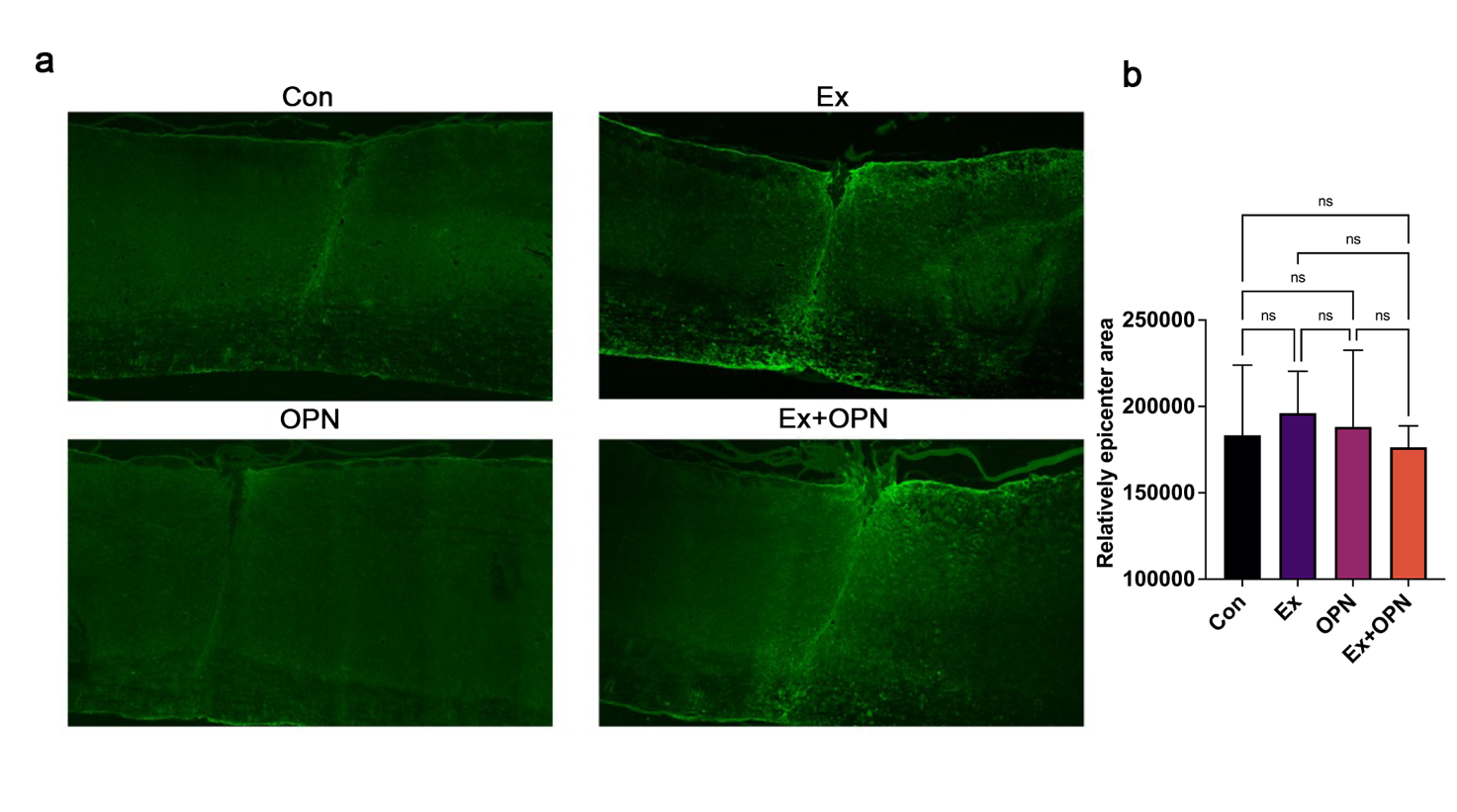
**

**Figure S2.** **epicenter area** (a)Representative fluorescence images of longitudinal spinal cord sections from the Con, Ex, OPN, and Ex+OPN groups showing GFAP (green). (b) Quantification of the epicenter area.

Supplementary figure 3


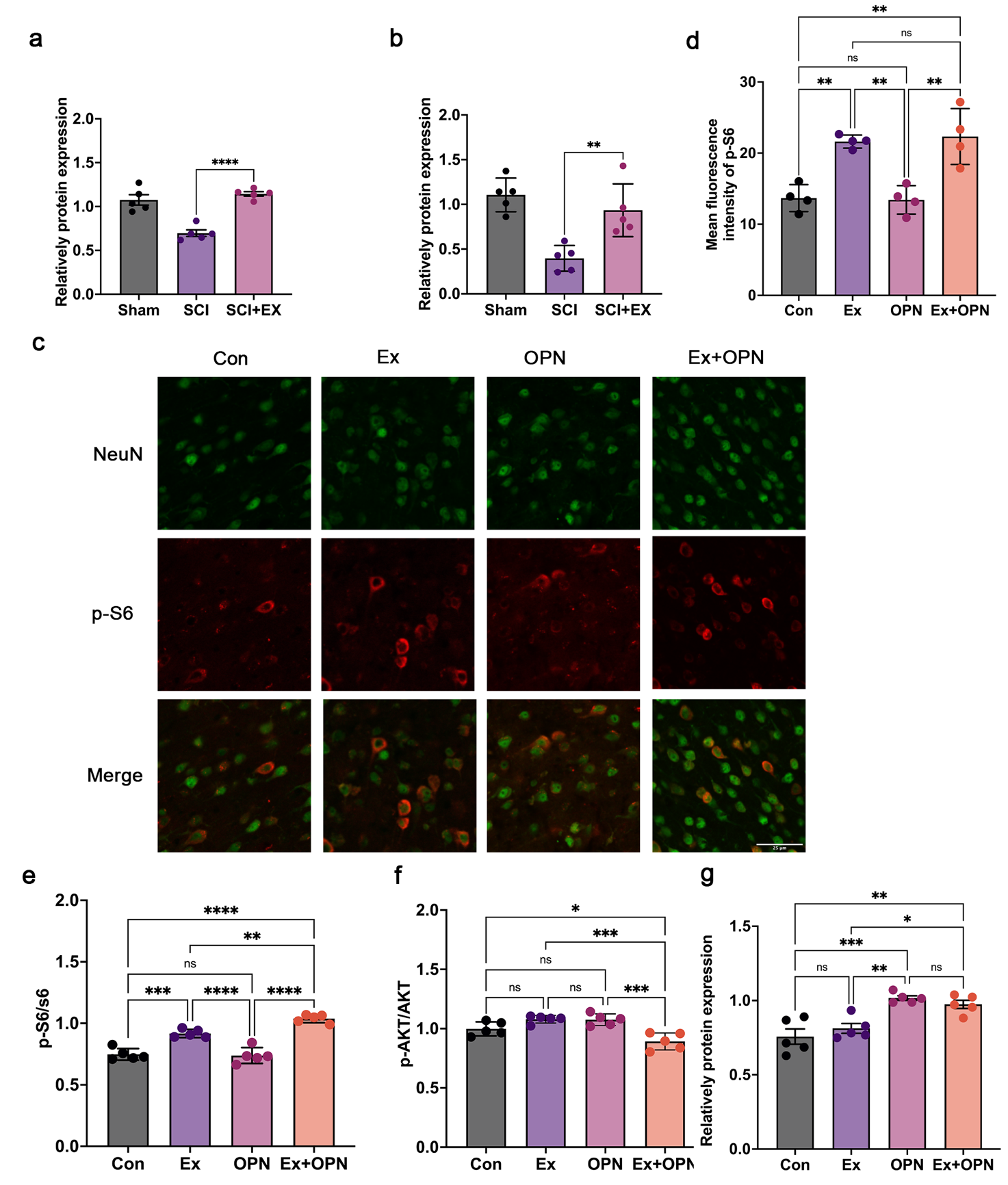


**Figure S3.** (a) The quantitative analysis of BDNF. (b) Quantitative analysis of IGF-1. (c) Representative fluorescence images of transverse brain sections for the Con, Ex, OPN, and Ex+OPN groups showing NeuN (green) and p-S6 (red). Bar = 25 μm.(d) Quantification of the p-S6 mean fluorescence intensity. (e) Quantitative analysis of p-S6/S6. (f) Quantitative analysis of p-AKT/AKT. (g) Quantitative analysis of IR.

Supplementary figure 4


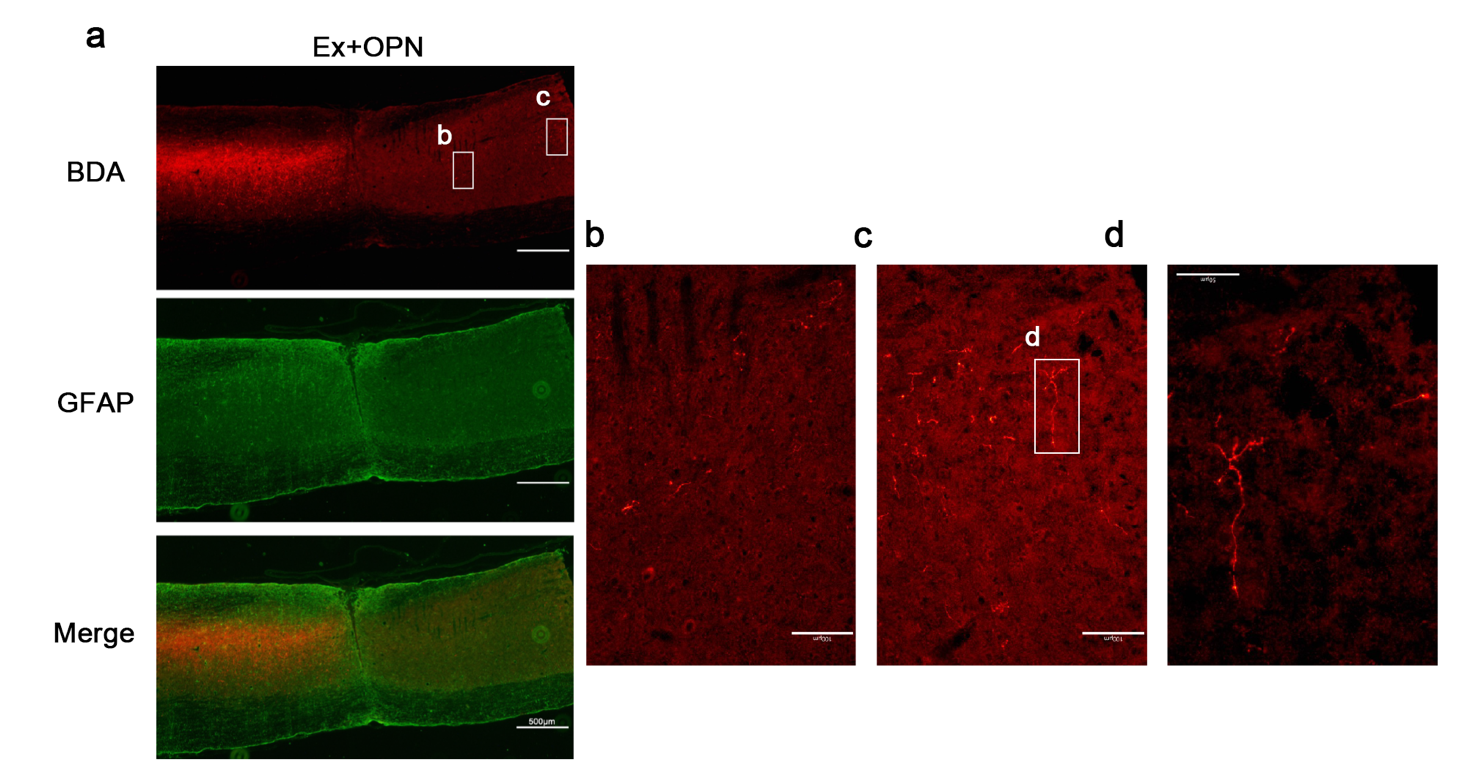


**Figure S4. Long-distance regeneration in the Ex+OPN group.** (a) Representative fluorescence images of longitudinal spinal cord sections from the Ex+OPN group. showing GFAP (green) and BDA (red). Bar = 500 μm. (b)(c) Magnification of the areas in the dotted box of A. Bar = 100 μm. (d) Magnification of the areas in the dotted box of c. Bar = 50 μm.
